# ERVK Polyprotein Processing and Reverse Transcriptase Expression in Human Cell Line Models of Neurological Disease

**DOI:** 10.3390/v7010320

**Published:** 2015-01-20

**Authors:** Mamneet Manghera, Jennifer Ferguson, Renée Douville

**Affiliations:** 1 Department of Biology, University of Winnipeg, Winnipeg, Manitoba R3B 2E9, Canada; E-Mails: sheena_m17@hotmail.com (M.M.); jennferguson@gmail.com (J.F.); 2 Department of Immunology, University of Manitoba, Winnipeg, Manitoba R3E 0T5, Canada

**Keywords:** endogenous retrovirus, reverse transcriptase, astrocyte, neuron, neurological disease, inflammation, IFNγ

## Abstract

Enhanced expression of the reverse transcriptase (RT) protein encoded by human endogenous retrovirus-K (ERVK) is a promising biomarker for several inflammatory and neurological diseases. However, unlike RT enzymes encoded by exogenous retroviruses, little work has been done to identify ERVK RT isoforms, their expression patterns, and cellular localization. Using Western blot, we showcase the ERVK gag-pro-pol polyprotein processing leading to the production of several ERVK RT isoforms in human neuronal (ReNcell CX) and astrocytic (SVGA) models of neuroinflammatory disease. Since the pro-inflammatory cytokine IFNγ plays a key role in the pathology of several ERVK-associated neurological diseases, we sought to determine if IFNγ can drive ERVK RT expression. IFNγ signalling markedly enhanced ERVK polyprotein and RT expression in both human astrocytes and neurons. RT isoforms were expressed in a cell-type specific pattern and the RT-RNase H form was significantly increased with IFNγ treatment. Fluorescent imaging revealed distinct cytoplasmic, perinuclear and nuclear ERVK RT staining patterns upon IFNγ stimulation of astrocytes and neurons. These findings indicate that ERVK expression is inducible under inflammatory conditions such as IFNγ exposure—and thus, these newly established *in vitro* models may be useful in exploring ERVK biology in the context of neuroinflammatory disease.

## 1. Introduction

Reverse transcriptase (RT) is the signature protein of retroviruses; however, for endogenous retrovirus-K (ERVK; alias HERV-K) there is limited knowledge regarding its RT isoforms, expression patterns and cellular localization in human health and disease. Despite evidence of enhanced ERV expression (ERVW, ERVH, ERVK, *etc.*) associated with several inflammatory and neurological diseases [1,2,3,4,5,6,7], few studies have sought to specifically examine ERVK polymerase (*pol*) gene and RT protein expression [8,9,10,11]. Elevated levels of ERVK RT have been observed in the cortical neurons of patients with Amyotrophic Lateral Sclerosis (ALS) [8]. This observation is consistent with the measurement of RT activity in the CSF and serum of individuals with ALS, at levels similar to those found in Human Immunodeficiency Virus (HIV) positive individuals [12]. ERVK RT is emerging as a promising prognostic biomarker in breast cancer [13]. Clearly, improved detection assays for ERVK RT expression are likely to be useful in other ERVK-associated diseases, including cancers [13], HIV infection [14,15], ALS [8], schizophrenia [16], rheumatic disease [17] and human prion disease [18]. Despite no known causal relationship between ERVK and human disease, pathological contributions of ERVK proteins continue to shape our understanding of complex disease processes [1,15,19,20,21].

ERVK (HML-2) encodes a reverse transcriptase enzyme with RNase H activity of approximately 65 kDa [22]. It is currently unclear if the active form of ERVK RT acts as a heterodimer—one monomer with an RNase H domain and the other without—as seen with other RT enzymes [23]. As with typical RT proteins, this enzyme contains a conserved LPQG motif and the catalytic YIDD motif [22]. The expression of ERVK RT is dependent on protease processing of the Gag-Pro-Pol polyprotein. Protease cleavage of the ERVK Gag precursor has recently been examined using recombinant constructs [24,25]; however, there is little known regarding the proteolytic processing of the entire Gag-Pro-Pol polyprotein *in situ*.

ERVK expression often occurs in diseases with inflammatory underpinnings. For example, ERVK is concomitantly expressed during HIV infection, both in the periphery and the central nervous system [15,26]. ERVK-specific T cells have been shown to secrete IFNγ in response to their cognate ligands [27,28]. Enhanced IFNγ levels in the brains of HIV-infected individuals [29], are believed to contribute to HIV-associated neuropathology [30]. Indeed, IFNγ has been shown to enhance HIV replication in astrocytes [31,32]. Therefore, we sought to stimulate human astrocyte and neuronal cell cultures with IFNγ, as a potential mechanism to drive ERVK RT expression. 

## 2. Materials and Methods

### 2.1. Cell Culture and Cytokine Treatment

The SVGA cell line [33] (gifted by Dr. Avindra Nath, NIH) is derived from immortalized human foetal astrocytes, and was maintained in Dulbecco’s modified Eagle’s medium supplemented with 10% Fetal Bovine Serum and 1% Penicillin/Streptomycin (HyClone, South Logan, UT, USA). ReNcell CX cells [34] (Millipore, Temecula, CA, USA) are immortalized human neural progenitor cells (HNPCs), and were maintained in a proprietary ReNcell neural stem cell medium (Millipore) supplemented with 20 ng/mL human epidermal growth factor (EGF; Peprotech, Rocky Hill, NJ, USA), 20 ng/mL human basic fibroblast growth factor (bFGF; Peprotech), and 1% Penicillin/Streptomycin. All cell lines were maintained in a humidified chamber containing 5% CO_2_ at 37 °C.

SVGA cells were seeded into six-well plates and onto glass coverslips in twelve-well plates at a density of 300,000 cells/mL and 30,000 cells/mL, respectively, and grown for 24 h. To differentiate HNPCs into neurons, ReNcells were seeded in laminin (20 μg/mL; Millipore) coated six-well plates at a density of 50,000 cells/mL for 24 h. Adhered cells were rinsed with 1X PBS and allowed to differentiate in the presence of ReNcell medium lacking EGF and bFGF for two weeks. SVGAs and neurons were treated with 0.1, 0.5, 1, and 5 ng/mL doses of human IFNγ (PeproTech) for 24 h. Plated untreated cells were used as negative controls.

### 2.2. Quantitative Polymerase Chain Reaction (Q-PCR)

Total RNA was extracted and purified from cells using an Aurum Total RNA Mini Kit (Bio-Rad, Hercules, CA, USA). RNA concentration was measured with a NanoDrop spectrophotometer. The acceptable RNA purity was A_260_/A_280_ > 2.0. The iScript Reverse Transcription kit (Bio-Rad) was used to synthesize cDNA from extracted RNA. CFX Connect Real Time System (Bio-Rad) was employed to perform Q-PCR in order to measure alterations in ERVK gag and pol transcripts using SYBR Green detection method. The primers used to amplify ERVK gag were F: 5' TCGGGAAACGAGCAAAGG 3' and R: 5' GAATTGGGAATGCCCCAGTT 3', and for ERVK pol were F: 5' TGATCCCMAAAGAYTGGCCTT 3' and R: 5' TTAAGCATTCCCTGAGGYAACA 3'. 18S rRNA was used as the endogenous control (Ambion, Carlsbad, CA, USA). The data was analysed using the ΔΔCT (Livak) method. GraphPad Prism [35] was used to carry out statistical analyses including column statistics, One-way Anova Friedman test, and Dunn’s post-test.

### 2.3. Reverse Transcriptase (RT) Assay

The activity of reverse transcriptase (RT) in protein fractions isolated from cells was measured using an EnzChek Reverse Transcriptase Assay Kit (Molecular Probes, Carlsbad, CA, USA), as per manufacturer’s instructions. Soluble and insoluble protein fractions were prepared at a fixed protein concentration, and pooled at a 1:1 ratio to perform each reaction. MMLV RT standards (Bio-Rad) were also run over a 4-log_10_ dilution series, and used to construct the standard curve. RT activity was quantitated by measuring the end point fluorescence of each reaction using CFX Connect Real Time System (Bio-Rad) and compared to that of the standard curve. GraphPad Prism [35] was used to carry out statistical analyses including column statistics, One-way Anova Friedman test, and Dunn’s post-test.

### 2.4. Western Blotting

Cells were lysed on ice with 50 μL of in-house lysis buffer (0.05 M Tris (pH 7.4), 0.15 M NaCl, 0.002 M EDTA, 10% glycerol and 1% NP-40 in ultra-pure water) to extract the soluble proteins, followed by extraction of insoluble proteins in 50 μL of RIPA buffer (10% 1X TBS, 1% SDS, 1% NP-40 and 0.5% DOC in ultra-pure water). Both buffers were supplemented with 1x HALT protease and phosphatase inhibitor cocktail (Thermo Scientific, Rockford, IL, USA). BCA assay (Thermo Scientific) was used to determine the protein content of each sample as per manufacturer’s instructions. Cell lysates were prepared for SDS-PAGE and heated at 95 °C for 10 min. Proteins (15 μg per lane) were separated by SDS-PAGE using a 10% polyacrylamide gel, and transferred onto a nitrocellulose membrane. The membrane was blocked in 5% skim milk solution for one hour and probed with mouse anti-human ERVK2 RT primary antibody (1:1000 dilution; Abnova, Jhongli City, Taiwan, ROC) overnight at 4 °C, followed by incubation at room temperature for 3 h. The membrane was then probed with horseradish peroxidase-conjugated goat anti-mouse IgG secondary antibody (1:5000 dilution; Bio-Rad) for 2 h at room temperature. β-actin was detected using mouse anti-human β-actin primary (1:5000 dilution; Thermo Scientific) and goat anti-mouse secondary antibodies, and was used as the loading control. The membrane was developed with 2 mL of Luminata Crescendo Western HRP substrate (Millipore) and imaged using Bio-Rad ChemiDoc XRS+ chemiluminescent imager. Image Lab software [36] was used to determine the molecular weight of each band, as well as their density relative to that of the negative control. The identity of each band was predicted based on the molecular weight of each ERVK protein [37] and informed by the gag-pro-pol processing pattern of HIV [38,39], including HIV protein post-translational modifications [40].

### 2.5. Fluorescent Imaging

Cells were fixed with methanol (Fisher Scientific, Fair Lawn, NJ, USA) for 1 min and rinsed with 1× PBS. Cells were permeabilised with 250 μL of PBS-T (PBS with 0.25% TritonX-100) and blocked with 250 μL of 3% BSA in TBS-T (TBS with 0.25% TritonX-100) for 30 min. Cells were incubated in primary antibodies (1:200 dilution) for one hour, followed by incubation in appropriate fluorophore-conjugated secondary antibodies (1:1500 dilution) for one hour. Mouse anti-human ERVK2 RT and rabbit anti-human α-tubulin (Abnova) were used as primary antibodies. Alexa Fluor 488 goat anti-mouse IgG (Molecular Probes) and Alexa Fluor 594 goat anti-rabbit IgG (Molecular Probes) were the secondary antibodies. Nuclei were counter-stained with DAPI (1:50,000 dilution; Molecular Probes). Controls were prepared by immunostaining without the primary antibodies. Coverslips with stained SVGAs were mounted onto slides using ProLong Gold anti-fade reagent (Molecular Probes). Confocal 2D images and 3-plane view images were acquired using an Olympus Fluoview FV1200 confocal microscope with the FV10-ASW4.0 software suite. Six-well plates with stained neurons were imaged using an EVOS FL Cell Imaging System (Life Technologies, Carlsbad, CA, USA). 

## 3. Results and Discussion

**Figure 1:**
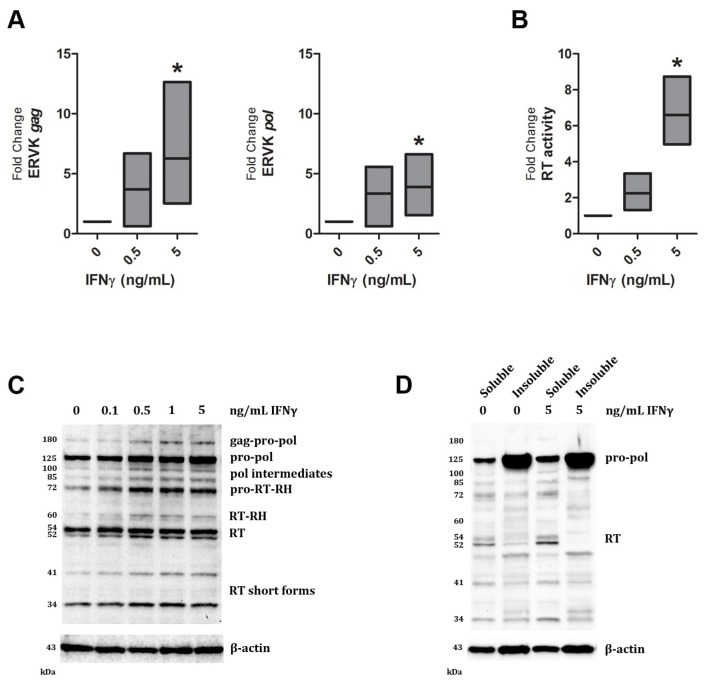
ERVK polyprotein and reverse transcriptase expression is inducible in IFNγ-treated astrocytes. The SVGA cell line was treated with increasing doses (0 to 5 ng/mL) of the cytokine IFNγ for 24 h. (**A**) IFNγ treatment enhances ERVK transcription, as measured by Q-PCR using *gag* and *pol*-specific primers (*n* = 5). * = *p* < 0.05; (**B**) IFNγ stimulation of astrocytes promotes elevated cellular RT activity (*n* = 4); * = *p* < 0.05 (**C**) Representative Western blot depicts proteins detected by a commercial anti-ERVK reverse transcriptase antibody (AbNova) or an anti-β-actin antibody control. IFNγ exposure enhances ERVK gag-pro-pol polyprotein (180 kDa), as well as several protease-cleaved forms of this viral polyprotein (*n* = 4). The two expected heterodimeric forms [46] of the ERVK RT are present in IFNγ-treated astrocytes; a 60 kDa form with an RNase H (RH) domain and a 52/54 kDa form without the RNase H domain. Several bands appear as doublets, such as the 52/54 kDa RT band, and likely represent post-translational protein modifications [40]. Short forms of the ERVK RT (41 and 34 kDa bands) may be truncated forms or represent instability and degradation of the RT protein [47]; (**D**) In both untreated and IFNγ-stimulated SVGA cells, the majority of the pro-pol polyprotein exists in an insoluble form within cells (*n* = 3). In contrast, the RT isoforms are concentrated in the soluble cytoplasmic fraction of the cell.

Augmented IFNγ signalling is a hallmark of several neurological diseases including ALS [41] and HIV-associated neuropathology [30]. Both exogenous (HIV) and endogenous (ERVW) retrovirus expression can be enhanced by IFNγ stimulation [7,31,32,42]. IFNγ is a potent activator of pro-inflammatory transcription factors Interferon Response Factor 1 (IRF1) and Nuclear Factor-kappa B (NF-κB), and can enhance HIV gene expression through interaction of these transcription factors with the HIV promoter [31,32,43]. Similarly, we have recently shown that the ERVK promoter also harbours multiple conserved putative binding sites for IRF1 and NF-κB [44], suggesting that IFNγ signalling may also enhance ERVK transcription and protein levels. In support of this evidence, Figure 1A shows that indeed ERVK transcription is enhanced upon IFNγ treatment of human astrocytes, perhaps through increased binding of NF-κB and IRF1 with the ERVK promoter. The levels of the ERVK *gag-pol* transcript were assessed by Q-PCR using *gag* and *pol*-specific primers. IFNγ treatment significantly enhanced ERVK transcription in a dose-dependent manner (5 ng/mL IFNγ, *p* < 0.05). In order to determine whether this transcriptional increase in ERVK expression was correlated with evidence of functional viral proteins, we also assessed overall RT activity in this model. Basal RT activity was observed in untreated SVGA cells; however, upon IFNγ treatment RT activity substantially increased (Figure 1B; 5 ng/mL IFNγ, *p* < 0.05). This method is unable to identify the viral source of the RT activity; therefore, we employed an ERVK RT-specific antibody to address whether ERVK polyprotein processing occurred, producing active RT isoforms, under inflammatory conditions. Figure 1C demonstrates that IFNγ is capable of enhancing ERVK polyprotein (gag-pro-pol, 180 kDa) and RT (60 and 52 kDa forms) expression in astrocytes. 

Similar to HIV polyprotein processing [38,39], multiple protease cleavage steps produce intermediate protein products, before each RT isoform is released from the polyprotein. Active RT enzymes are generally heterodimers comprised of a large catalytic RT isoform containing an RNase H domain and a smaller RT isoform without the RNase H domain, which plays a structural role [40,45,46]. Figure 1C shows the formation of two different sized ERVK RT isoforms. The short ERVK RT form of 52/54 kDa is expressed at basal levels in astrocytes, and dose-dependently increases with IFNγ treatment (Table 1), with an optimal stimulating dose of 0.5 ng/mL of IFNγ. Of note, ERVK RT bands appear as doublets, suggesting that they may be post-translationally modified, as seen with HIV-1 RT phosphorylation [40]. The 60 kDa ERVK RT-RNaseH isoform is expressed only upon IFNγ stimulation, suggesting that RT activity may optimally occur under inflammatory conditions, such as low-level chronic IFNγ exposure. Additionally, the appearance of active and structural ERVK RT isoforms leads us to propose that ERVK may be a cellular source of RT activity in inflammatory disease. Figure 1D demonstrates that the majority of the ERVK polyprotein (pro-pol form) is found in the insoluble fraction of the SVGA whole cell lysate, whereas the RT proteins were found within the soluble cytoplasmic fraction. 

**Table 1:** Fold change in ERVK polyprotein and RT band intensity normalized to β-actin loading control for Western blot in Figure 1A.

Band size (kDa)	IFNγ dose (ng/mL)				
	0	0.1	0.5	1	5
180	1.0	0.7	2.6	2.6	2.6
125	1.0	1.1	1.9	1.5	3.0
100	1.0	1.1	2.4	1.8	2.0
85	1.0	2.0	3.9	3.5	4.2
72	1.0	1.8	2.7	2.4	2.6
60	1.0	1.9	2.8	1.7	2.0
54	1.0	1.2	1.5	1.1	1.2
52	1.0	1.8	2.5	1.8	2.1
41	1.0	1.4	2.2	2.4	2.8
34	1.0	1.1	1.6	1.4	1.2

**Figure 2:**
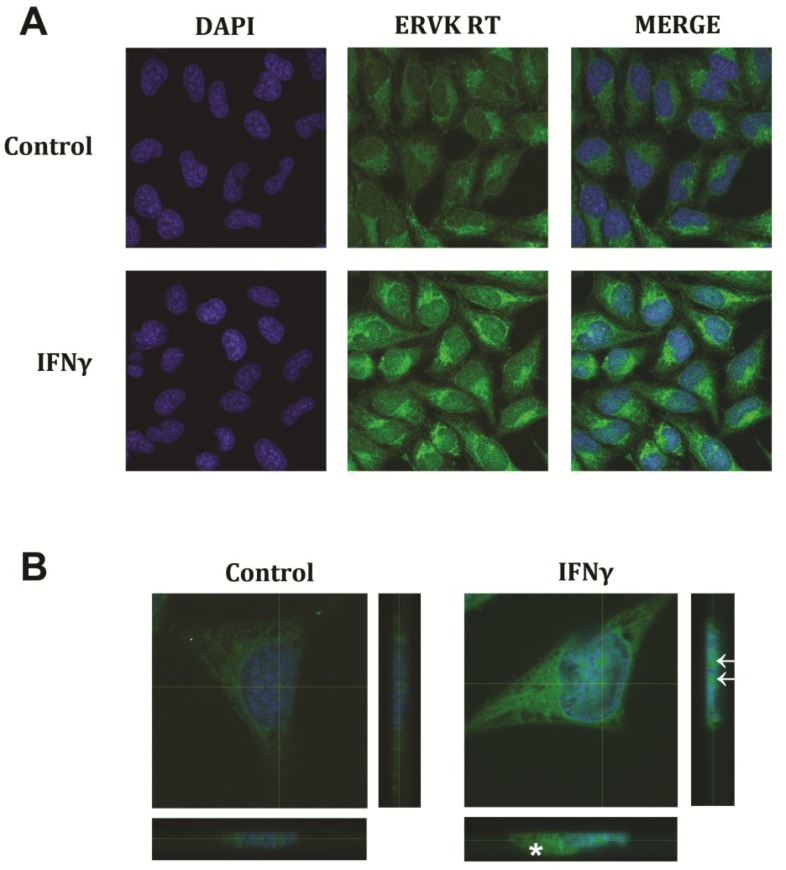
IFNγ enhances ERVK RT expression in human astrocytes. The SVGA cell line was treated with 5 ng/mL of IFNγ for 24 h. Cells were immunostained using a commercial anti-ERVK reverse transcriptase primary antibody (Abnova) and fluorescently-labelled secondary antibody. Nuclei were stained with DAPI. Images were acquired using an Olympus FV1200 laser scanning confocal microscope. (**A**) Representative micrographs show basal ERVK RT staining in untreated astrocytes, while IFNγ-stimulated cells exhibit a substantial increase in ERVK RT staining as compared to the control. Magnification 200X; (**B**) Untreated and IFNγ treated astrocytes were evaluated using a 9 μm Z-stack (0.5 μm steps) which depicts ERVK RT expression throughout the entire cell. 3D projections (X, Y and Z planes of crosshair sections) confirm enhanced cytoplasmic, perinuclear and nuclear (arrows) ERVK RT staining in IFNγ treated astrocytes. Accumulation of ERVK RT in IFNγ treated cells is associated with cellular swelling (asterisk). Magnification 600X.

The anti-ERVK RT antibody was also used to perform fluorescent immunocytochemistry on SVGA cells. Based on the Western blot data from Figure 1, we expect that the ERVK RT staining pattern represents the sum of intracellular polyprotein and RT isoforms. IFNγ-mediated ERVK RT expression was observed in the cytoplasm, with a non-uniform distribution (Figure 2A). ERVK proteins may act similarly to HIV, whereby the large subunit of RT or the polyprotein can interact with β-actin [48]. RT-actin interactions are known to be a fundamental and dynamic process in reverse transcription and localization of reverse transcription complexes (RTCs) [49]. ERVK RT expression also accumulated around the nucleus (Figure 2A,B), as observed with HIV-1 RTCs [50]. The formation of a perinuclear ring with a large RT protein aggregate proximal to the nucleus occurs concurrently with cellular swelling. Nuclear ERVK RT expression exhibited a speckled pattern (Figure 2B), and may reflect nuclear import of RTCs [50].

**Figure 3:**
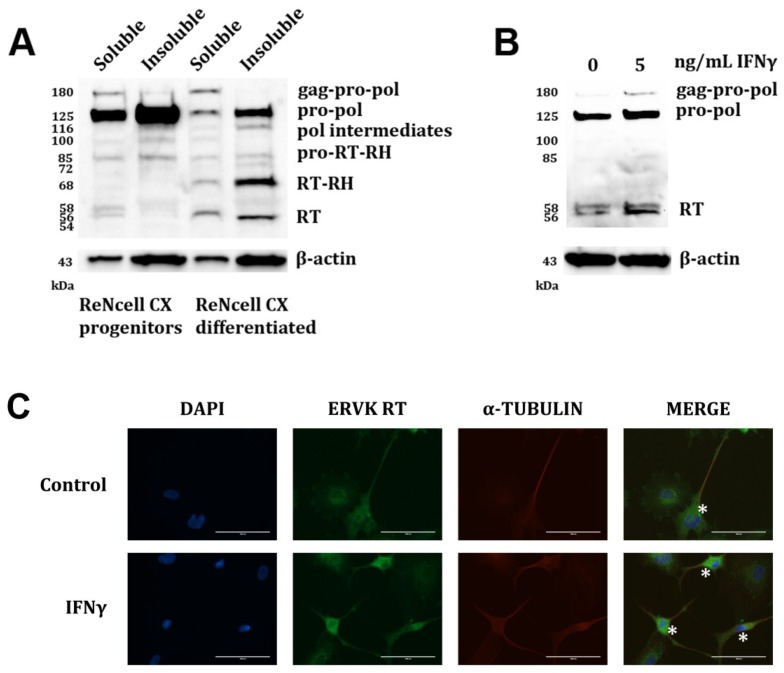
IFNγ enhances ERVK polyprotein and RT expression in human neurons. Representative Western blots depict proteins detected by a commercial anti-ERVK reverse transcriptase antibody (Abnova) and an anti-β-actin antibody control. (**A**) Soluble and insoluble cell fractions of ReNcell CX progenitors and ReNcell CX neurons differentiated by growth factor withdrawal. Differentiated neurons express enhanced ERVK gag-pro-pol polyprotein (180 kDa), RT-RH (68 kDa) and RT (56/58 kDa) isoforms as compared to ReNcell progenitors (*n* = 2); (**B**) ReNcell CX-derived neurons were treated with 5 ng/mL IFNγ for 24 h (*n* = 1). IFNγ exposure enhances ERVK gag-pro-pol polyprotein (180 kDa), as well as RT levels in soluble whole cell lysates; (**C**) ReNcell CX-derived neurons were treated with 5 ng/mL IFNγ for 24 h (*n* = 3). Cells were immunostained using anti-ERVK reverse transcriptase primary antibody (Abnova), anti α-tubulin primary antibody (Abnova), and fluorescently-labelled secondary antibodies. Nuclei were stained with DAPI. Images were acquired using an EVOS FL microscope. Representative micrographs show basal ERVK RT staining in untreated neurons (asterisk) and glial cells, while IFNγ-stimulated neurons (but not glia) exhibit a substantial increase in ERVK RT staining in the cell body as compared to the control. Images acquired using a 40X objective.

In ALS [8] and HIV infection [15], ERVK expression occurs in cortical neurons. We have employed ReNcell CX neural progenitor cell line [34] as a means to study ERVK expression in human neurons. Figure 3A demonstrates that these progenitor cells exhibit enhanced ERVK polyprotein expression; however, differentiation of these neural progenitor cells through growth factor deprivation substantially reduces ERVK pro-pol polyprotein levels (>10-fold decrease) and promotes the expression of RT (10-fold increase) in the soluble fraction. ERVK RT isoforms in ReNcell cultures exhibited increased mass as compared to SVGAs (RT-RH 68 kDa *versus* 60 kDa and RT 56/58 kDa *versus* 52/54 kDa, Figure 3 *versus* Figure 1, respectively), and may represent cell-type specific post-translational modification of RT [40]. For example, HIV RT is phosphorylated at several sites [51], suggesting that our data also may depict several phosphorylated forms of ERVK RT. The cell-type specific differences in RT isoform mass may be related to differential capacity for phosphorylation patterns in neurons *versus* astrocytes [52]. Treatment of these neuronal cultures with IFNγ also enhances the expression of the 180 kDa ERVK polyprotein and 56 kDa RT from that of basal levels (2.6 fold and 2.4 fold, respectively) (Figure 3B). Figure 3C shows that within the differentiated ReNcell culture, IFNγ treatment promotes ERVK RT expression in neuronal cells, but not glial cells. Basal ERVK RT expression was evident in untreated neurons; however, ERVK RT expression was markedly enhanced in the cell body of IFNγ-treated neurons. This model of enhanced ERVK RT in the neuronal cell body is consistent with the immunoreactive staining pattern observed in the cortical brain tissue of patients with ALS [8].

For the first time, we show that the pro-inflammatory cytokine IFNγ can enhance ERVK polyprotein expression and promote its cleavage into heterodimeric RT isoforms. These working *in vitro* models of RT expression in astrocytes and neurons will permit the further examination of ERVK biology in the context of inflammatory neurological disease.
